# Developing a Fluorescence-Based High-Throughput Screening Method for Natural Product Fractions to Identify Inhibitors of AR-V7 for Castration-Resistant Prostate Cancer

**DOI:** 10.3390/pharmaceutics18091158

**Published:** 2026-09-15

**Authors:** Neha Tyagi, Manish Rathi, Susan Ensel, Christopher C. Thornburg, Tanja Grkovic, Barry R. O’Keefe, James C. Sacchettini, Chendil Damodaran

**Affiliations:** 1 Department of Pharmaceutical Sciences, Texas A&M University, College Station, TX 77843, USA; neha.tyagi@tamu.edu; 2 Department of Biochemistry and Biophysics, Texas A&M University, College Station, TX 77843, USA; manish.rathi@ag.tamu.edu; 3 Natural Products Support Group, Leidos Biomedical Research Inc., Frederick National Laboratory for Cancer Research, Frederick, MD 21702, USA; 4 Department of Chemistry and Physics, Hood College, Frederick, MD 21701, USA; 5 Natural Products Branch, Developmental Therapeutics Program, Division of Cancer Treatment and Diagnosis, National Cancer Institute, Frederick, MD 21702, USA; 6 Molecular Targets Program, Center for Cancer Research, National Cancer Institute, Frederick, MD 21702, USA

**Keywords:** natural products, androgen receptor, AR-V7, castration-resistant prostate cancer, high-throughput screening, HiBiT reporter, butanolides

## Abstract

**Background/Objectives:** The progression of prostate cancer to castration-resistant prostate cancer (CRPC) is often driven by constitutively active androgen receptor splice variants, such as AR-V7, which evade conventional androgen-deprivation therapies. This study aimed to develop a high-throughput, mechanism-based screening platform to identify natural-product-derived AR-V7 inhibitors from the NCI Program for Natural Product Discovery (NPNPD) library. **Methods:** A subset of prefractionated NPNPD samples was screened using a CRISPR-edited 22Rv1 cell line expressing endogenous AR-V7 fused to a HiBiT luminescent tag, enabling quantification of AR-V7 levels. Fractions that reduced the HiBiT signal were further evaluated in 22Rv1 and C4-2B CRPC cells and counter-screened in non-malignant RWPE-1 cells. Compounds demonstrating at least 90% inhibition in CRPC cells with no more than 10% toxicity in RWPE-1 cells underwent dose–response analysis, Western blotting, quantitative PCR, and subfractionation to isolate and characterize active constituents. **Results:** Of the 704 prefractionated samples, five fractions met stringent activity and selectivity criteria, with three consistently suppressing full-length androgen receptor (AR), AR-V7, and prostate-specific antigen (PSA). Primary screening results in HiBiT-22Rv1 cells were validated by secondary dose–response and Western blot assays, and hits were further prioritized to ensure toxicity remained at or below 10% in RWPE-1 cells. HiBiT-guided screening of 66 subfractions identified seven that robustly downregulated AR signaling, with reduced AR-V7 levels correlating with decreased CRPC cell viability. Chemical characterization revealed two active butanolides, isolitsealiicolide C and isolinderanolide B, which reduced AR and AR-V7 protein and mRNA levels, decreased PSA, downregulated Bcl-2, and induced cleaved PARP, consistent with apoptotic cell death in AR-positive CRPC models. **Conclusions:** This integrated high-throughput workflow efficiently identifies AR-V7–targeted natural products from complex libraries and highlights isolitsealiicolide C and isolinderanolide B as promising scaffolds for overcoming androgen receptor–driven resistance in CRPC.

## 1. Introduction

Prostate cancer (PCa) is the most frequently diagnosed malignancy and a leading cause of cancer-related mortality among men globally [1,2]. Although advances in early diagnosis and treatment have been made, projections for the United States estimated 313,780 new cases and 35,770 deaths in 2025, with recent data indicating that this burden will persist in 2026 [3,4]. Uncontrolled activation of the androgen receptor (AR) is recognized as a primary driver for the initiation and progression of PCa [5]. Consequently, androgen deprivation therapy (ADT) has become the cornerstone of PCa treatment, achieved either by reducing circulating testosterone or by binding to AR and suppressing AR signaling [6]. Despite the effectiveness of ADT, approximately 10–20% of men with PCa progress to castration-resistant prostate cancer (CRPC), which constitutes the lethal stage of the disease [7,8].

Multiple resistance mechanisms, including mutations in the AR ligand-binding domain (LBD) and the emergence of constitutively active AR-splice variants (AR-SVs), contribute to the progression of CRPC [9]. AR-SVs are truncated forms of the AR that lack the LBD but retain other functional domains, rendering current ADT ineffective and leading to disease progression and recurrence [10]. Structural and biophysical studies indicate that the AR-N-terminal domain (NTD) is highly dynamic and contains unconventional binding pockets, which complicates efforts to target this domain [11,12,13]. As a result, high-throughput screening (HTS) approaches based on traditional structure-based design have been developed to identify more potent AR LBD-targeting compounds, given their commercial viability and translational potential [14,15]. Nevertheless, the AR-NTD remains a challenging and largely unaddressed therapeutic target.

To date, only a limited number of AR-NTD-directed inhibitors have been identified, including the ralaniten (EPI-001/EPI-506) series, the sintokamides, and the niphatenones, all of which originate from marine or plant natural product sources [16,17]. Molecular studies indicate that these inhibitors downregulate the expression of both full-length AR and AR-SVs, thereby inhibiting CRPC growth. Efforts have also focused on repurposing existing drugs; for example, the anthelmintic agent niclosamide suppresses the expression and activity of the AR splice variant AR-V7 with minimal impact on full-length AR [18]. Additionally, numerous research groups have identified natural compounds that modulate AR signaling [19,20]. However, the clinical translation of these agents has been limited by high-dose requirements, poor bioavailability, and challenges in isolation and large-scale production. Semi-synthetic modification of natural compounds may enhance potency, improve pharmacokinetic properties, and facilitate manufacturing [21,22,23]. Nevertheless, no approved therapies currently exist that effectively inhibit AR-V7 in CRPC, leaving many patients with AR-SV–driven CRPC reliant on palliative treatment options and highlighting the critical need for novel strategies targeting this domain.

Nearly 60% of approved anticancer agents are natural products or their direct derivatives, such as taxanes, vinca alkaloids, camptothecins, and anthracyclines [24,25,26,27,28,29]. These findings highlight the significance of natural products as a viable source for anticancer drug discovery, with substantial translational impact. However, natural products that bind to and modulate AR-NTD remain largely unexplored. Thus, it is essential to identify AR-V7 inhibitors using broader screening technologies and more diverse compound libraries beyond currently available cutting-edge robotic molecular tools. Advances in targeted assays and HTS platforms have facilitated progress in precision oncology by enabling rapid identification of new active molecules and structurally modified compounds across diverse cancer models [30]. The NCI Program for Natural Product Discovery (NPNPD) has the largest publicly available library of prefractionated natural product samples derived from plants, marine invertebrates, and microorganisms. These resources have already yielded inhibitors targeting p38 and TDP1 among several other molecular targets for cancer [31,32,33]. These successes suggest that such libraries may also contain compounds capable of inhibiting AR-NTD while exhibiting favorable drug-like properties.

Building on this rationale, the present study aims to develop a cell-based HTS platform to screen the NPNPD prefractionated natural product library against AR-V7 in CRPC. A novel approach was developed using a CRISPR-edited 22Rv1 cell line, in which elevated endogenous AR-V7 activity was tagged with a HiBiT peptide to enable luminescent readouts for natural product screening. After identifying fraction hits in the primary screen, subfractionation was conducted as previously described to isolate the most potent pure or semi-purified natural products [34]. Active compounds were subsequently isolated and validated in additional AR-positive CRPC cell lines and non-cancerous prostate epithelial cells. This strategy combines the established advantages of natural products in cancer research with modern screening methods to identify new compounds targeting the AR-NTD. These findings may inhibit both ligand-dependent and ligand-independent AR signaling. They also provide alternatives to conventional LBD antagonists and lay the groundwork for future research on clinically relevant NTD-directed inhibitors using the screening technology developed in this study.

## 2. Materials and Methods

### 2.1. Cell Lines and Reagents

The human prostate cancer cell lines 22Rv1 and C4-2B, as well as the non-malignant prostate epithelial cell line RWPE-1, were obtained from the American Type Culture Collection (ATCC, Manassas, VA, USA) and authenticated by short tandem repeat (STR) analysis. To identify potential AR-V7 inhibitors, a CRISPR-edited 22Rv1 cell line expressing endogenous AR-V7 fused to a HiBiT tag, an 11-amino-acid peptide that binds a complementary inactive luciferase subunit (LgBiT), was purchased from Promega (Madison, WI, USA; Item # CS3023258; AR(v7)-HiBiT KI 22Rv1). The addition of LgBiT and substrate generated a luminescent signal proportional to AR-V7 levels in 22Rv1 cells. The AR-HiBiT knock-in 22Rv1 cell line was obtained from Promega, which provides authentication and quality-control documentation for genome-edited reporter lines. All cell stocks were maintained according to the providers’ recommendations and used within the passage ranges specified by ATCC and Promega.

All cells were cultured in RPMI medium supplemented with 10% fetal bovine serum (FBS) and 1× penicillin–streptomycin [35]. RWPE-1 cells were maintained in Keratinocyte Serum Free Medium (K-SFM; Invitrogen/GIBCO, Grand Island, NY, USA, Kit Catalog Number 17005-042) supplemented with bovine pituitary extract (BPE) and human recombinant epidermal growth factor (EGF), according to the manufacturer’s instructions. All experiments used 22Rv1, C4-2B, and RWPE-1 cells between passages 3 and 5, with passage numbers kept constant across replicates to minimize experimental variation.

### 2.2. NPNPD Natural Product Library

The NPNPD natural product library was supplied in 384-well plates, with each well containing 10 µg of a single fraction dissolved in 4 µL of DMSO (2.5 µg/µL). Library stocks were serially diluted 5-fold and then 4-fold to generate working solutions corresponding to final assay concentrations of 50, 25, 10, 5, 2.5, and 1.25 µg/mL. All concentrations were initially screened, and 1.25 µg/mL was selected as the optimal screening concentration based on assay performance, dynamic range, and primary hit rate. Compounds were transferred by adding 0.5 µL of the working solution to a final assay volume of 50 µL, resulting in a final DMSO concentration of 1% (*v*/*v*). Control experiments confirmed that 1% DMSO did not affect cell viability.

### 2.3. Cybio Liquid Handling Platform for HTS

High-throughput screening (HTS) of natural product fractions was performed using the Cybio liquid handling platform (Analytik Jena, Jena, Germany), a fully automated, high-precision pipetting system optimized for miniaturized assay formats. The system was configured for 384-well plates, enabling accurate and reproducible dispensing of nanoliter- to microliter-volume compounds, reagents, and detection substrates. NPNPD samples were diluted in DMSO and dispensed into 384-well plates using the Cybio platform to ensure uniform delivery and minimize cross-contamination. The platform was also used for automated addition of assay components, including resazurin for cell viability measurements and Nano-Glo HiBiT Lytic Detection Reagent for luminescence-based assays.

### 2.4. Selection of Cytotoxicity Assay and HTS Conditions

To minimize interference from intrinsically fluorescent natural products, resazurin, MTT (3-[4,5-dimethylthiazol-2-yl]-2,5-diphenyltetrazolium bromide), and CellTiter-Glo luminescent viability assays were compared for cytotoxicity measurements. The resazurin assay exhibited the highest robustness and reproducibility and was therefore selected for high-throughput screening (HTS) optimization. Cell density optimization was performed by plating between 0 and 500,000 cells/mL in 384-well plates (Advanced TC, 384-well microplate, black, clear bottom with lid (Greiner Bio-One, Monroe, NC, USA, Cat# 781986), 89131-696; Supplementary Figure S1). The minimum cell density that generated a reproducible, non-saturated resazurin signal was 10,000 cells/mL, corresponding to 500 cells in 50 µL per well. Cells were seeded at this density and cultured to 60–75% confluency prior to treatment. Plates were treated with vehicle control (DMSO; Sigma-Aldrich, St. Louis, MO, USA), positive control (withaferin A), or test samples from challenge plates (0.5–1 µL/well) and incubated at 37 °C with 5% CO_2_ for 24 h. A 24 h exposure was chosen as a standardized time point for primary screening to capture early cytotoxic effects and maintain a consistent assay window across all fractions. This approach facilitated comparability and minimized the advancement of marginal hits to secondary studies. Following treatment, 22Rv1, C4-2B, and RWPE-1 cells were stained with resazurin (0.2 mg/mL) and incubated at 37 °C. Fluorescence was measured at Ex/Em 560/590 nm after a 16 h incubation. IC_50_ values for prioritized fractions and pure compounds were calculated using CompuSyn software (Version: 1.0.1) [36]. For HiBiT-based quantification of AR-V7, AR–HiBiT–expressing 22Rv1 cells were plated at 10,000 cells/mL in opaque white 384-well plates and at 3000 cells/well in 96-well white plates. These two formats were implemented for distinct stages of the workflow: 384-well plates facilitated high-throughput primary screening, whereas 96-well plates were utilized for confirmatory and mechanistic assays that required larger signal windows and greater flexibility for replicate measurements. Cell densities in each format were independently optimized to achieve comparable luminescence within the linear detection range and to maintain similar levels of confluency at the 24 h endpoint. Key findings were reproduced across both formats, and conditions were standardized within each assay type to minimize variability in compound responses.

### 2.5. AR–HiBiT Quantification

The effects of test compounds on AR–HiBiT-tagged protein levels were quantified using the Nano-Glo HiBiT Lytic Detection System (Promega, Cat# N3040). AR–HiBiT-expressing cells were plated at 10,000 cells/mL in opaque white 384-well tissue-culture-treated plates (Greiner Bio-One, Monroe, NC, USA, Cat# 781095) and at 3000 cells/well in 96-well white opaque tissue-culture-treated plates (Pierce, Waltham, MA, USA, Cat# 15042) to minimize optical crosstalk and maximize luminescence detection. These conditions were optimized independently, and key findings were validated across both plate formats to reduce variability. After 24 h of compound treatment, luminescence was measured using a microplate reader. Parental 22Rv1 cells lacking the HiBiT knock-in were used as HiBiT-negative controls. Background luminescence was determined from negative control wells and subtracted from all readings to yield the specific HiBiT signal.

### 2.6. Western Blot Analysis

22Rv1 and C4-2B cells were exposed to the IC_50_ concentrations of NPNPD fraction hits or purified compounds. Protein extraction and Western blotting followed previously described protocols [37]. Primary antibodies targeting AR (#5153, Cell Signaling Technology, Danvers, MA, USA), PSA (#5365S, Cell Signaling Technology), Bcl-2 (#15071S, Cell Signaling Technology), PARP (#9542, Cell Signaling Technology), and β-actin (#5125, Cell Signaling Technology) were employed, along with HRP-conjugated secondary antibodies (#7074S and #7076S, Cell Signaling Technology). The AR antibody recognizes total AR and detects both full-length AR and the AR-V7 splice variant as separate bands on immunoblots. Blots were visualized using a ChemiDoc system (Bio-Rad, Hercules, CA, USA), and band intensities were quantified by densitometry using ImageJ 1.54p.

### 2.7. qPCR Analysis

Total RNA was extracted from 22Rv1 and C4-2B cells treated with either vehicle or isolinderanolide B for 6 h using the RNeasy Mini Kit (Qiagen, Hilden, Germany) according to the manufacturer’s instructions. DNase I treatment (Cat# EN0521; Thermo Fisher Scientific, Waltham, MA, USA) was performed to eliminate genomic DNA contamination. RNA concentration and purity were measured using a NanoDrop spectrophotometer, and RNA integrity was confirmed prior to cDNA synthesis. Equal amounts of total RNA (1 µg) were reverse-transcribed using iScript Reverse Transcription Supermix (Cat# 1708840; Bio-Rad).

qPCR was conducted using SsoAdvanced Universal SYBR Green Supermix (Cat# 1725270; Bio-Rad) on a CFX96 Touch Real-Time PCR Detection System (Bio-Rad). Cycling conditions were as follows: polymerase activation at 95 °C for 30 s, denaturation at 95 °C for 10 s, annealing/extension at 60 °C for 20 s for 40 cycles, and a melt curve from 65 °C to 95 °C in 0.5 °C increments.

Primer sequences were as follows: AR (forward, 5′-TCAGACAGTCAAGAATTTCAGAGC-3′; reverse, 5′-CGCACAGGTACTTCTGTTTCC-3′), AR-V7 (forward, 5′-TGAAGCAGGGATGACTCTGG-3′; reverse, 5′-TCAGCCTTTCTTCAGGGTCTG-3′), PSA (forward, 5′-CCCACTGCATCAGGAACAA-3′; reverse, 5′-ATATCGTAGAGCGGGTGTGG-3′), and β-actin (forward, 5′-CTCCTCCACCTTTGACGCTG-3′; reverse, 5′-CATACCAGGAAATGAGCTTGACAA-3′). β-actin served as the reference gene for normalization. Each reaction included three biological replicates and technical triplicates.

### 2.8. Collection, Extraction, and Isolation

*L. glutinosa* was collected in Vietnam and taxonomically identified by Dr. Djaja D. Soejarto (University of Illinois at Chicago) under contract with the Natural Products Branch, National Cancer Institute. A voucher specimen (0GHA1453) was deposited at the Smithsonian Institution, Washington, DC. Dried and ground stem wood (584 g) was extracted with MeOH/DCM (1:1) to yield 15.31 g of crude organic extract (N137135). A 1 g portion of this extract was pre-fractionated on C8 solid-phase extraction (8 g) by sequential elution with H_2_O/MeOH (95:5, 38.2 mg, fraction 1), H_2_O/MeOH (80:20, 58.5 mg, fraction 2), H_2_O/MeOH (60:40, 18.9 mg, fraction 3), H_2_O/MeOH (40:60, 67.2 mg, fraction 4), H_2_O/MeOH (20:80, 170.1 mg, fraction 5), MeOH (168.7 mg, fraction 6), and MeOH/MeCN (50:50, 152.6 mg, fraction 7).

Fraction 6 (168 mg) was further separated by reversed-phase HPLC on a Phenomenex (Torrance, CA, USA) Onyx monolithic C18 column (100 × 10 mm) at 3.8 mL/min using the following gradient: 70% H_2_O (0.1% formic acid, FA)/30% MeCN (0.1% FA) from 0 to 1.5 min (isocratic) and a linear gradient to 100% MeCN (0.1% FA) over 7.5 min, followed by an isocratic hold at 100% MeCN (0.1% FA) for 3.5 min. For each run, 5 mg of material was injected, and 22 fractions were collected in 30 s intervals from 1.3 to 12.3 min. Fractions 18 and 20 were further purified on the same column at 3.8 mL/min with 50% H_2_O/50% MeCN (0 to 5 min, isocratic), a linear gradient to 100% MeCN over 25 min, and an isocratic hold at 100% MeCN for 10 min to yield isolitsealiicolide C (1.0 mg, 0.6% yield) and isolinderanolide B (0.8 mg, 0.5% yield).

### 2.9. Isolation of Pure Compounds

Isolitsealiicolide C was obtained as a clear oil, with chirooptical and NMR spectroscopic data consistent with literature values: HRESIMS *m*/*z* [M + H]^+^ 281.2110 (calculated for C_17_H_29_O_3_^+^ 281.2106) [38]. Isolinderanolide B was also obtained as a clear oil, with chirooptical and NMR spectroscopic data consistent with literature values [39]: HRESIMS *m*/*z* [M + H]^+^ 309.2433 (calculated for C_19_H_33_O_3_^+^ 309.2419).

Statistical analysis. Quantitative data for confirmatory cell viability and qPCR experiments were obtained from at least three independent biological replicates. Technical replicates were incorporated as specified for each assay. For qPCR, each biological replicate was analyzed in technical triplicate. Data are presented as mean ± standard deviation (SD), unless otherwise indicated in the figure legends. Cell-viability signals were normalized to vehicle-treated controls and reported as either percent viability or percent inhibition. *p*-values are indicated as follows: *p* < 0.05 (ns), *p* < 0.05 (*), *p* < 0.01 (**), *p* < 0.001 (***), and *p* < 0.0001 (****).

Initial screening of natural-product extracts employed either a resazurin-based cell-viability assay or a HiBiT luminescence assay. The NPNPD library was supplied in blinded 384-well plates. High-throughput screening performance was assessed using the Z′ factor. Following unblinding, fractions were designated as active hits if they resulted in at least 90% inhibition in CRPC cells, no more than 10% inhibition in RWPE-1 cells, and a Z-score greater than 3 relative to the plate mean. Half-maximal inhibitory concentration (IC50) values were determined by nonlinear regression analysis of dose–response curves using CompuSyn software. Statistical comparisons between vehicle- and compound-treated groups were performed using an unpaired two-tailed Student’s *t* test for two-group comparisons, or one-way analysis of variance (ANOVA) with an appropriate multiple-comparisons test for experiments involving more than two groups. A two-sided *p*-value less than 0.05 was considered statistically significant. Western blot quantification was performed using ImageJ software.

## 3. Results

A validated HTS platform was established using a subset of the NPNPD prefractionated library, consisting of 704 natural product fractions distributed across two 384-well challenge plates. Prefractionated and characterized NPNPD samples exhibit reduced chemical complexity compared to traditional crude extracts, thereby facilitating subsequent isolation and structural elucidation of active compounds [40,41]. The 704 fractions were assessed in AR/AR-V7-positive CRPC cell lines 22Rv1 and C4-2B. RWPE-1 normal prostate epithelial cells served as a toxicity counter-screen to identify fractions that selectively target CRPC while sparing non-malignant prostate cells.

**Figure 1:**
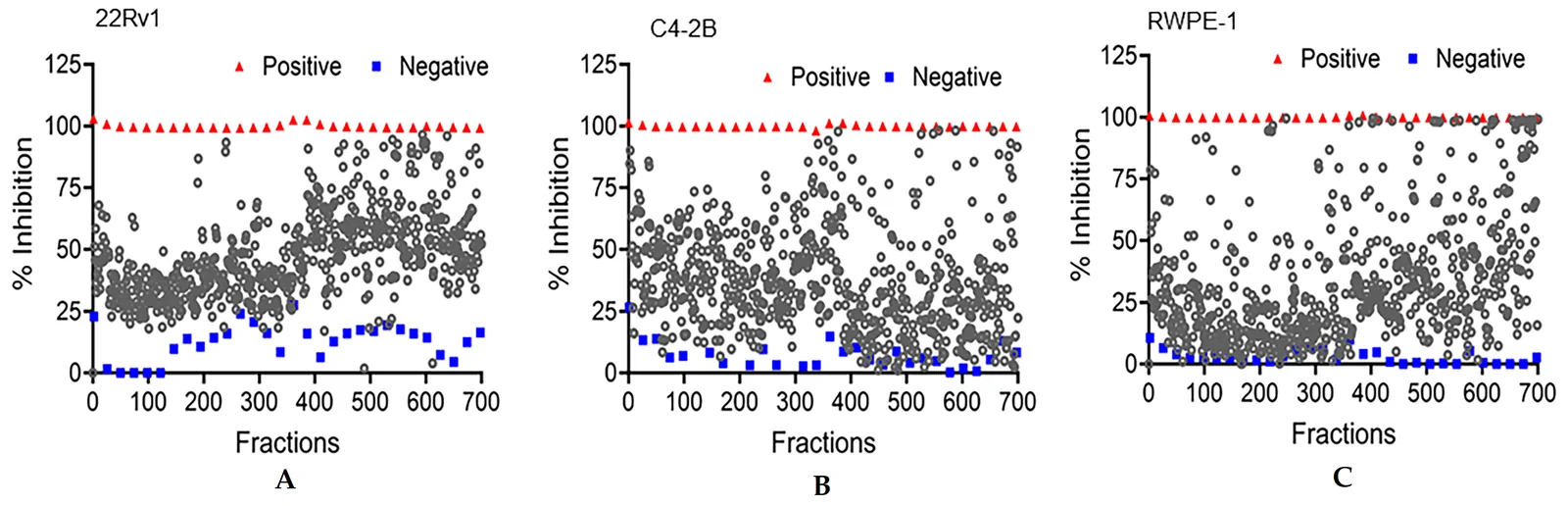
High-throughput screening of natural product fractions identifies selective inhibitors of CRPC cell growth. (**A**–**C**) Cell viability was measured using a 384-well resazurin-based assay. Results are shown as percentage viability relative to the control group, from which percentage inhibition was calculated. The CRPC cell lines 22Rv1 (**A**) and C4-2B (**B**), as well as the non-malignant prostate epithelial cell line RWPE-1 (**C**), were evaluated. Viability was determined by quantifying fluorescence from the reduced resazurin metabolite. Blue squares indicate negative controls, red triangles indicate positive controls, and gray circles indicate the screened fractions. The assay demonstrated robust performance, with an average plate Z′ factor of 0.6, supporting reliable hit identification.

To minimize interference from intrinsically fluorescent fractions, multiple cell viability assays were compared, including resazurin, MTT, and CellTiter-Glo. The resazurin assay demonstrated superior robustness, reproducibility, and minimal signal interference and was therefore selected for HTS optimization [42]. Cell density for each CRPC line (22Rv1 and C4-2B) and test concentrations (25, 10, 5, 2.5, and 1.25 µg/mL) were systematically optimized (Supplementary Figure S2; Figure 1A–C). Analysis of viability data indicated that a final test concentration of 1.25 µg/mL provided an optimal dynamic range and a primary hit rate of approximately 1.4%. This concentration was chosen as a practical compromise that maintained sensitivity to active fractions while limiting the number of marginal hits, consistent with recommendations for HTS of complex natural product libraries [40]. In parallel, we established a stringent activity threshold, defining hits as samples that produced ≥90% growth inhibition in 22Rv1 and C4-2B cells, which enriched for strongly cytotoxic and mechanistically relevant fractions and reduced the likelihood of advancing weak or nonspecific inhibitors. For all screening experiments, withaferin A, a natural compound previously shown to inhibit CRPC growth, was used at 15 µM as a positive control to benchmark assay performance [43].

An active hit was defined as a fraction that produced at least 90% inhibition of 22Rv1 and C4-2B cell viability, no more than 10% inhibition in RWPE-1 cells, and a z-score greater than 3 above the plate mean. The 90% inhibition cutoff served as a stringent criterion to enrich for fractions with robust and reproducible activity, while minimizing the progression of weak or borderline effects. During assay optimization, lower thresholds, such as 50–70% inhibition, substantially increased the number of marginal hits that were not consistently confirmed in secondary dose–response and Western blot assays. In contrast, a cutoff of at least 90% inhibition at the optimized screening concentration of 1.25 µg/mL yielded a manageable hit rate, provided clear separation from plate noise, and aligned with recommendations for high-stringency phenotypic screening of complex natural product libraries.

### 3.1. Secondary Validation and Mechanism-Focused Assays

**Figure 2:**
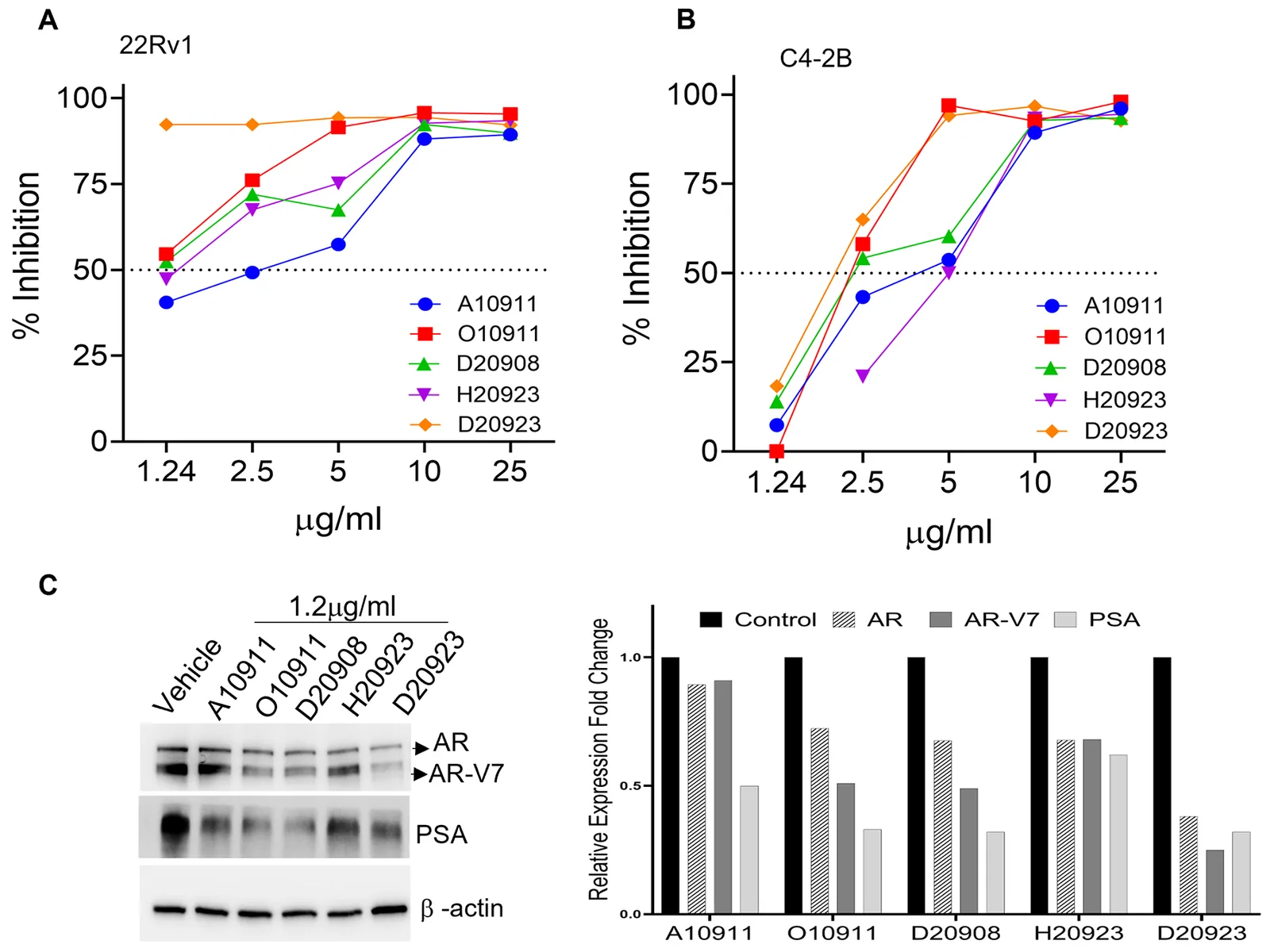
Validation of natural product fractions demonstrates dose-dependent cytotoxicity and downregulation of AR signaling. 22Rv1 (**A**) and C4-2B (**B**) cells were treated with increasing concentrations of selected fractions for 24 h, and cell viability was quantified using a resazurin-based assay. Results are shown as percentage viability relative to the control group, from which percentage inhibition was calculated. Data are reported as mean ± SD from triplicate wells. The assay maintained consistent performance, with an average Z′ factor of 0.6. (**C**) 22Rv1 cells were treated with the indicated crude fractions at their respective IC50 concentrations for 24 h. Whole-cell lysates were analyzed by Western blot to assess protein levels of full-length AR (AR-FL), AR-V7, and the downstream marker PSA, with β-actin as a loading control. Relative expression levels were quantified using ImageJ and normalized to β-actin (right).

The five most active fractions identified in the primary screen underwent further evaluation by dose–response analysis in 22Rv1 and C4-2B cells to determine inhibitory concentrations (IC_50_) (Figure 2A,B). Four fractions demonstrated IC_50_ values near 0.125 mg/mL, while one fraction exhibited limited activity in 22Rv1 cells and was excluded from subsequent mechanistic studies. IC_50_ concentrations were subsequently employed to assess AR signaling via Western blot analysis. Three fractions (010911, D20908, and H20923) substantially suppressed full-length AR, AR-V7, and PSA expression (Figure 2C). In contrast, the remaining two fractions displayed strong cytotoxicity toward 22Rv1 and C4-2B cells but did not significantly inhibit AR-V7 or PSA, indicating potential off-target or AR-independent mechanisms of cell death.

Of the five prioritized fractions, 010911, D20908, and H20923 consistently achieved approximately 90% growth inhibition in 22Rv1 and C4-2B cells, while maintaining toxicity at or below 10% in RWPE-1 cells. These fractions satisfied the combined criteria for potency, AR-axis modulation, and selectivity and were therefore advanced to subfractionation. This process provided a targeted starting point for deconvoluting complex mixtures into individual bioactive entities, consistent with the established NPNPD workflow [34]. The three lead fractions underwent semi-preparative HPLC subfractionation, yielding 66 subfractions using the automated, high-capacity procedure developed by the NPNPD [41]. This methodology expedites the production of assay-ready subfractions, reduces chemical complexity prior to structural elucidation, and facilitates bioassay-guided isolation of active molecules.

### 3.2. Subfractionation and HiBiT–AR-V7 Reporter-Based Screening

To improve AR-V7-specific activity, the workflow was refined by incorporating a CRISPR-engineered 22Rv1-HiBiT cell line into the primary screening process, where endogenous AR-V7 was tagged with the HiBiT peptide [44]. This strategy enabled high-throughput luminescent quantification of AR-V7 abundance and transitioned the pipeline from a phenotypic screen to a mechanism-enriched platform [45]. All 66 subfractions derived from the three lead fractions were assessed in parallel using 22Rv1-HiBiT cells. Orthogonal viability assays were performed in parental 22Rv1 and C4-2B cells to confirm that reductions in HiBiT signal were associated with decreased CRPC cell viability.

**Figure 3:**
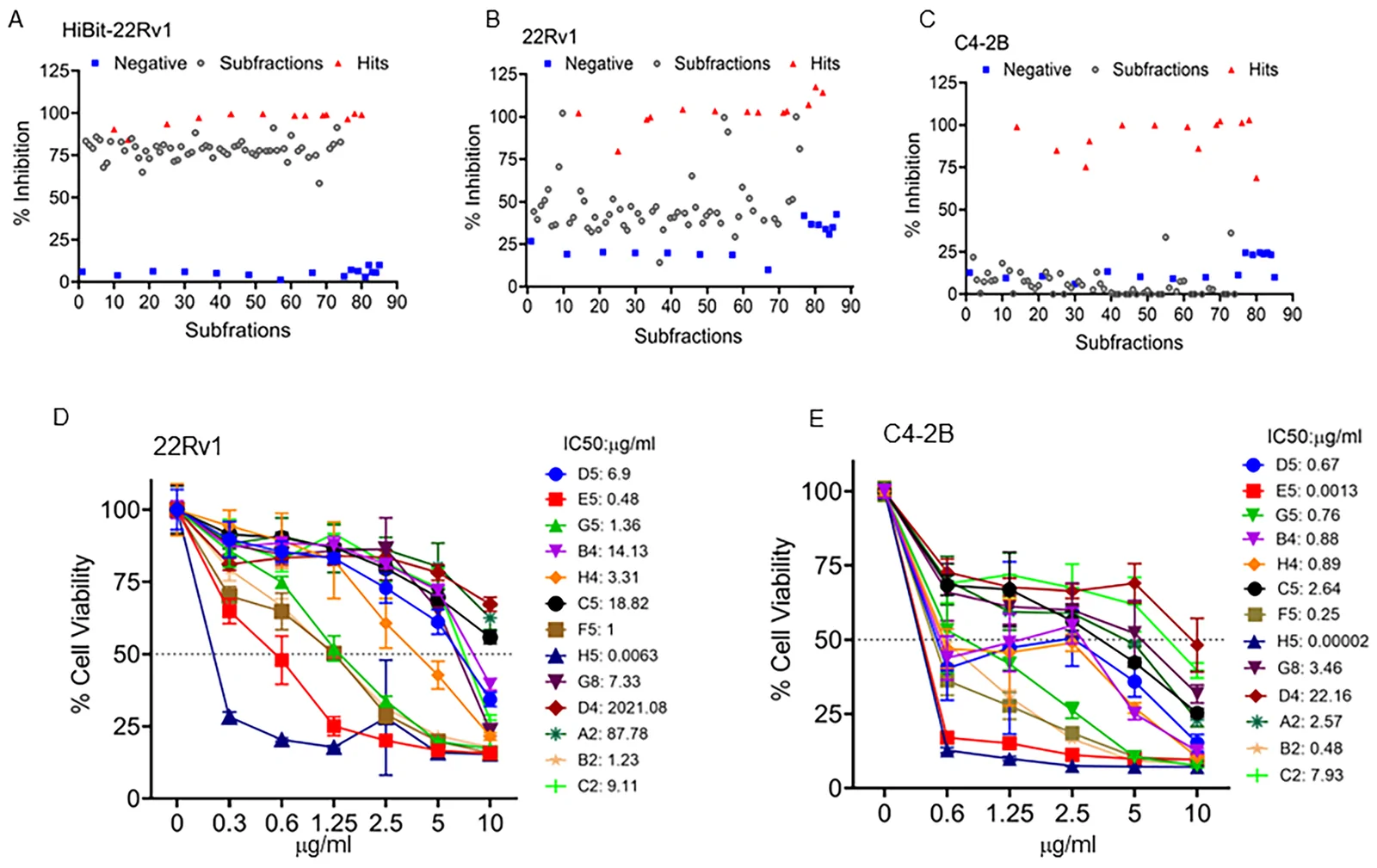
Identification of potent sub-fractionated fractions. (**A**) Primary screening of sub-fractionated NCI–NPNPD extracts was conducted in HiBiT–22Rv1 cells treated for 24 h to identify inhibitors of AR-V7 expression. (**B**) 22Rv1 and (**C**) C4-2B cells were treated with the same sub-fractionated fractions, and cell viability was measured using a resazurin-based assay after 24 h. Results are shown as percentage viability relative to the control group, from which percentage inhibition was calculated. Blue squares represent negative controls, red triangles represent positive controls, and gray circles represent the screened fractions. Dose–response effects of selected sub-fractions on cell viability were further evaluated in 22Rv1 (**D**) and C4-2B (**E**) cells using the resazurin assay after 24 h of treatment.

Based on the aforementioned active hit screening criteria, thirteen of the 66 subfractions exhibited inhibition across all three cell lines (22Rv1-HiBiT, 22Rv1, and C4-2B) (Figure 3A–C), demonstrating strong antiproliferative effects. These 13 subfractions were subsequently subjected to dose-dependent inhibition studies in both 22Rv1 and C4-2B cells (Figure 3D,E). Subfractions failing to meet stringent potency or selectivity criteria were excluded from further analysis. This iterative process ensured that only subfractions with consistent, mechanism-linked activity advanced in the workflow.

### 3.3. Validation and Identification of Lead Structures

**Figure 4:**
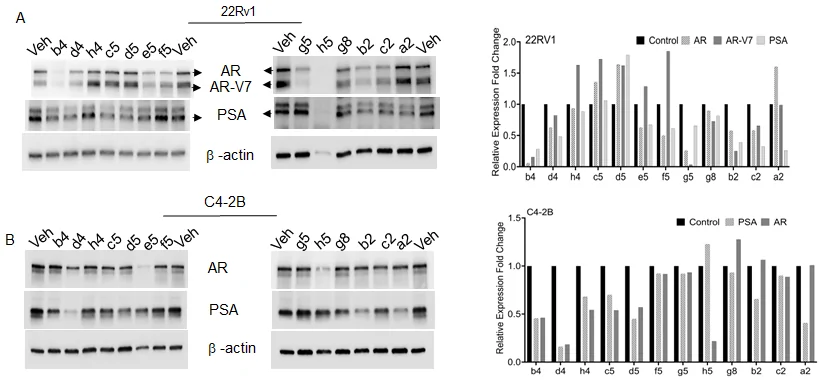
Tertiary screening confirms suppression of AR signaling by selected sub-fractionated fractions. Western blot analysis was conducted to evaluate the effects of the top 13 sub-fractions on AR signaling in CRPC cell lines. (**A**) 22Rv1 and (**B**) C4-2B cells were treated with the indicated fractions, and whole-cell lysates were analyzed for AR, AR-V7, and the downstream target PSA, with β-actin serving as a loading control. Relative expression levels were quantified using ImageJ and normalized to β-actin.

To elucidate the mechanism of action, the 13 most active subfractions were analyzed by Western blot in 22Rv1 and C4-2B cells. Seven subfractions revealed significant downregulation of both full-length AR and AR-V7, accompanied by decreased PSA expression (Figure 4A,B). These findings confirm functional inhibition of AR signaling and reinforce the mechanistic association between AR-axis suppression and reduced CRPC cell viability. The strong concordance between HiBiT reporter signal and phenotypic viability effects demonstrates that the HiBiT platform is a reliable primary filter for identifying subfractions that reduce AR-V7 levels and inhibit CRPC proliferation. These results further support AR-V7 as a functional driver of survival in this context, consistent with prior AR-V7 literature [46,47], and corroborate the viability data.

**Figure 5:**
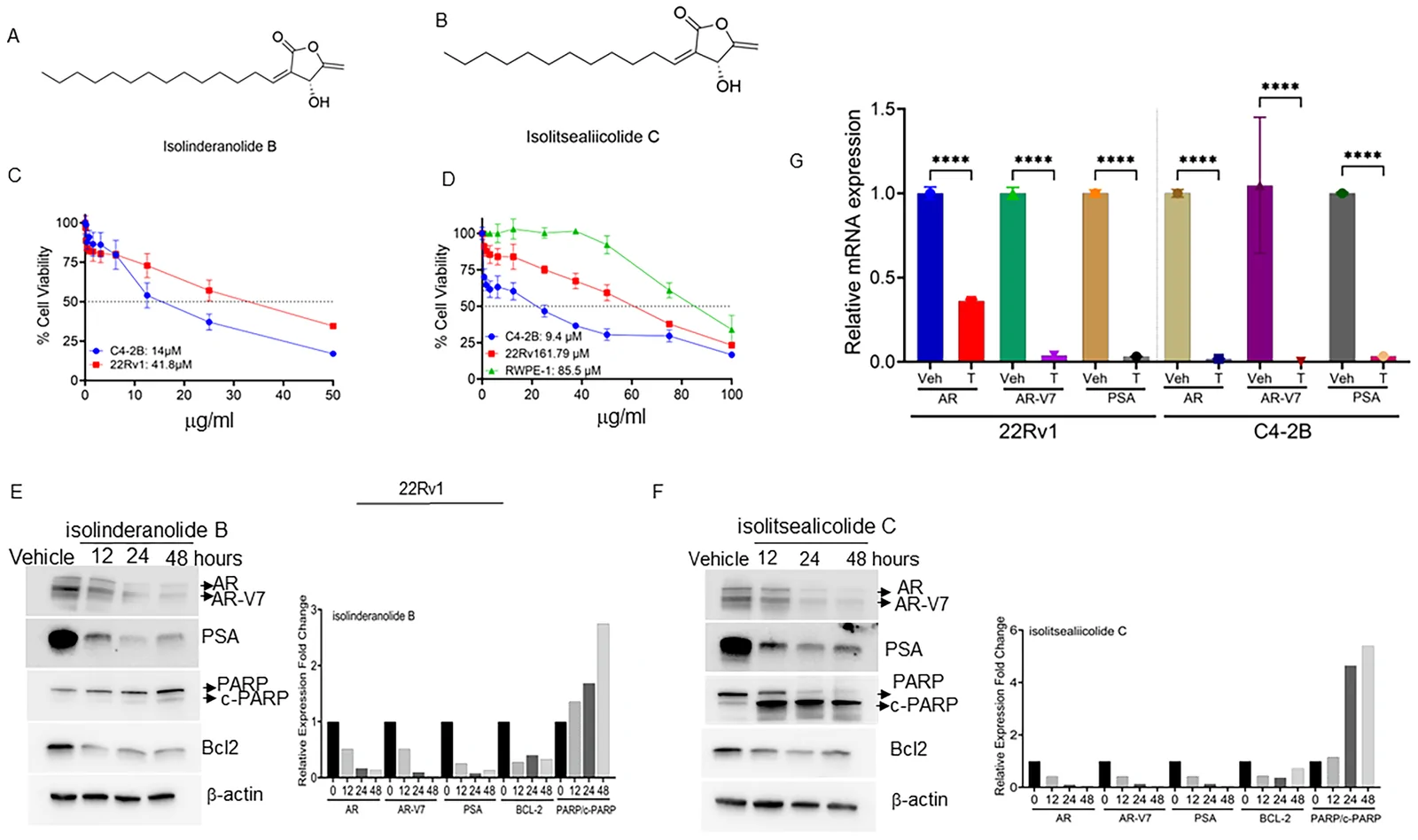
Identification and characterization of bioactive butanolides isolated from active sub-fractions. Chemical structures of two potent compounds, isolinderanolide B (**A**) and isolitsealiicolide C (**B**), are shown. Cells were treated with increasing concentrations of isolinderanolide B (**C**) or isolitsealiicolide C (**D**) for 24 h, followed by assessment of cell viability. Results are shown as percentage viability relative to the control group, from which percentage inhibition was calculated. (**E**) Western blot analysis of 22Rv1 cells treated with the IC50 concentrations of each compound at specified time points revealed decreased levels of AR, AR-V7, and PSA, as well as downregulation of Bcl-2 and induction of cleaved PARP for both compounds: isolinderanolide B (**E**) and isolitsealiicolide C (**F**). Relative expression levels were quantified by ImageJ and normalized to β-actin. (**G**) qPCR analysis of 22Rv1 and C4-2B cells treated with vehicle or isolitsealiicolide C for 6 h demonstrated reduced levels of AR, AR-V7, and PSA. *p* < 0.0001 (****).

Based on potency and selectivity, defined as robust AR-V7 inhibition with minimal RWPE-1 toxicity, seven subfractions were prioritized for chemical characterization. Using established NPNPD workflows, two known butanolides, isolitsealiicolide C and isolinderanolide B, were isolated and identified as major active principles (Figure 5A,B). Both compounds exhibited significant cytotoxicity toward 22Rv1 and C4-2B cells. Isolitsealiicolide C inhibited proliferation with IC_50_ values of 9.4 µM in C4-2B and 61.79 µM in 22Rv1 cells. In contrast, isolinderanolide B showed IC_50_ values of 14 µM in C4-2B and 41.8 µM in 22Rv1 cells (Figure 5C,D), indicating differential sensitivity across CRPC models. We also determined the toxicity of Isolitsealiicolide C on RWPE-1 cells as a proof-of-principle study and the IC_50_ concentration (85.5 mM) as compared to CRPC cells (Figure 5D). Consistent with these phenotypic effects, both compounds downregulated full-length AR, AR-V7, and PSA expression in 22Rv1 cells (Figure 5E,F). Quantitative PCR (qPCR) was conducted at the 6 h time point to evaluate early transcriptional responses to AR/AR-V7 pathway inhibition, prior to the onset of extensive apoptosis that could confound mRNA measurements. The observed rapid decrease in full-length AR and AR-V7 transcript levels, as well as PSA mRNA expression (Figure 5G), aligns with the established roles of AR/AR-V7 as transcriptional regulators. These findings indicate that the butanolides suppress AR-dependent gene expression; in parallel, the observed reduction in AR and AR-V7 protein levels supports an effect on the AR/AR-V7 signaling axis. Concurrently, Western blot analyses revealed upregulation of the pro-apoptotic marker cleaved poly(ADP-ribose) polymerase (PARP), a hallmark of caspase-dependent apoptosis, and downregulation of the pro-survival protein Bcl-2 (Figure 5E,F). Collectively, these data indicate that inhibition of AR and AR-V7 by these butanolide compounds disrupts AR-driven pro-survival signaling, shifts the balance of Bcl-2 family proteins toward apoptosis, and activates the mitochondrial caspase cascade, resulting in apoptotic cell death.

### 3.4. Validation Using Marine-Derived Fractions

**Figure 6:**
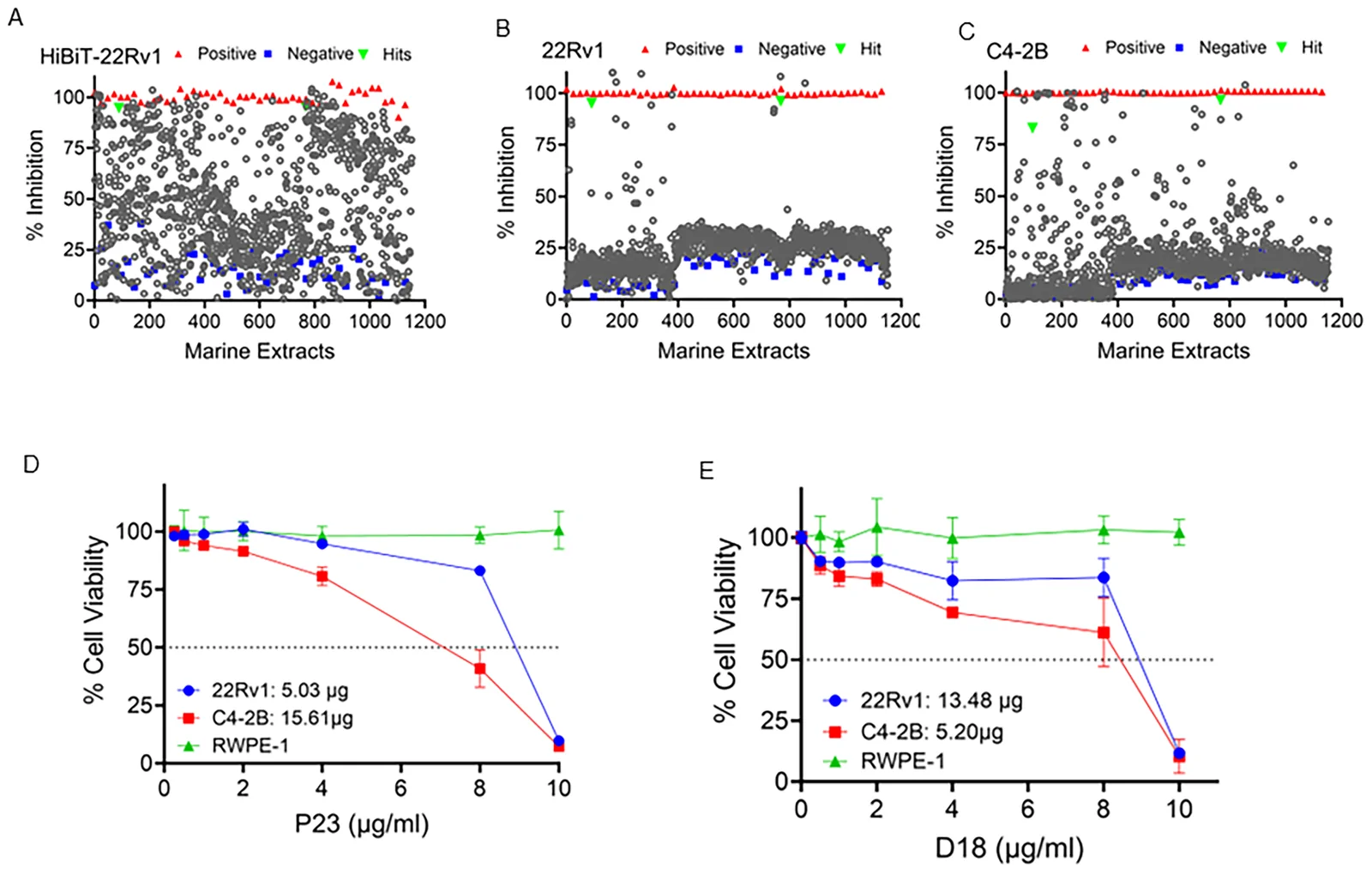
Screening and characterization of marine-derived fractions targeting AR-V7 in CRPC cells. (**A**) Primary screening of marine natural product fractions was conducted in HiBiT–22Rv1 cells to identify inhibitors of AR-V7 expression. The selected extract was evaluated for cytotoxicity in 22Rv1 (**B**) and C4-2B (**C**) cells using a resazurin-based assay. Blue squares indicate negative controls, red triangles indicate positive controls, and gray circles indicate the fractions screened. The two selected fractions, p23 and D18, were tested in 22Rv1, C4-2B, and RWPE-1 cells with increasing concentrations of the prioritized extract. Percentage growth inhibition relative to the vehicle control was calculated to determine the IC50 values for each cell line (**D**,**E**).

To evaluate the robustness and scalability of the optimized screening workflow, an expanded panel of 2816 additional marine-derived fractions from the NPNPD library was screened in 22Rv1-HiBiT, 22Rv1, and C4-2B cells (Figure 6A–C). Fractions were first assessed in 22Rv1-HiBiT cells to identify those that reduced AR-V7 abundance, with subsequent confirmation of activity in parental 22Rv1 and C4-2B cells. Two fractions were selected as proof of principle for toxicity and dose–response analysis. Fraction p23 exhibited IC_50_ values of 5.03 µg/mL in 22Rv1 and 15.61 µg/mL in C4-2B cells. In contrast, fraction D18 yielded IC_50_ values of 13.48 µg/mL and 5.20 µg/mL in 22Rv1 and C4-2B cells, respectively (Figure 6D,E). Both p23 and D18 were non-toxic to RWPE-1 cells, confirming their selectivity for CRPC models (Figure 6D,E). Comprehensive dose–response profiling, toxicity evaluation, subfractionation, and purification of all confirmed hits will be conducted in future studies. The overall confirmed hit rate in this larger marine subset was approximately 0.1%, aligning with expectations for stringent, mechanism-anchored screening of complex natural product libraries.

## 4. Discussion

In this study, the HTS technique was standardized to identify novel AR-V7 inhibitors from crude fractions using primary, secondary, and tertiary screening methods, yielding two compounds that inhibited the growth of CRPC. Traditional crude-extract screens are more complex and contain a greater diversity of compounds, making the isolation of active principles more difficult. In contrast, standardized NPNPD fractions facilitate the subsequent isolation of active compounds [40,41]. In our assays, we used a resazurin-based cell viability assay, which provided more reliable results in natural product screening than other assays that often require multiple steps before plate reading or may be affected by natural product fractions [42]. This homogeneous, no-wash assay eliminates the need for washing steps, thereby reducing manual handling and increasing efficiency. Furthermore, the method enabled screening of larger extract volumes during technique validation [48].

To streamline the workflow and reduce screening steps, the HiBiT-22Rv1 cell line was used for primary screening. This strategy enabled the identification of fractions that inhibit AR-V7 expression and suppress CRPC cell growth. Fractions with positive HiBiT assay results but lacking confirmation by secondary assays, such as Western blot, were excluded from further analysis. The HiBiT system is recognized as an effective tool for identifying AR-V7-targeted compounds [45]. Genome-edited reporter cell line technology is widely used in small-molecule screening [44]. The present study demonstrates that prefractionated libraries or complex fractions can be efficiently screened, potentially accelerating the discovery of natural products with defined mechanisms of action. This approach enabled high-throughput luminescent quantification of AR-V7 abundance and shifted the workflow from a purely phenotypic to a mechanism-enriched screening strategy [45].

Identifying small molecules that target AR-V7 is challenging because the AR N-terminal domain (AR-NTD) is disordered, hindering computational structural analyses of AR-V7 [49]. To address this challenge, several HTS methodologies have been employed to identify novel agents that modulate AR signaling, including measuring AR nuclear fluorescence intensity [50] and evaluating AR-V7 promoter activity [48]. HiBiT results were validated by Western blot analysis for AR, AR-V7, and its downstream gene PSA, a marker of the prostate gland; elevated PSA expression indicates prostate cancer [51]. AR-V7, unlike the full-length receptor, is continuously localized to the nucleus [51], and its expression correlates with androgen-independent cell proliferation and prostate cancer progression [52]. These findings provide a novel starting point for identifying agents that specifically inhibit AR-V7 expression in CRPC. However, the AR N-terminal domain remains a challenging target for current therapies. As AR-V7 variants confer resistance to androgen deprivation agents such as enzalutamide and abiraterone, identifying small molecules that reduce AR-V7 expression may help delay or overcome drug resistance in advanced prostate cancer [53]. The described workflow, which integrates multiple screening and validation techniques, could be adapted to address other challenging targets and facilitate the discovery of new molecules for proteins considered difficult to modulate. Because the AR N-terminal domain is intrinsically disordered and lacks stable binding pockets, AR-V7 inhibitors are difficult to design. However, natural products have demonstrated value against intrinsically disordered targets [11,54].

The identification of isolitsealiicolide C and isolinderanolide B as suppressors of both AR and AR-V7 highlights the effectiveness of the optimized stepwise screening approach. These compounds inhibited both the transcriptional and translational activity of AR, AR-V7, and PSA expression and induced markers of cell death, consistent with previous observations for lactones [55,56]. The purified compounds exhibited higher IC_50_ values in normal RWPE-1 cells than in CRPC cells, suggesting a preferential anticancer activity toward CRPC cells. However, toxicity may occur at higher concentrations of these compounds. Parent crude extracts showed limited toxicity toward normal prostate cells, but this may not apply to purified subfractions or individual compounds. Purification can concentrate specific bioactive components and change their activity or toxicity compared to the original extract. Therefore, the cytotoxicity, selectivity, and therapeutic window of each purified active compound should be independently assessed in non-tumor prostate cells over a broader concentration range in future studies.

The differences in IC_50_ values between 22Rv1 and C4-2B cells may reflect differences in AR signaling and other cell line-specific factors. Both cell lines are metastatic CRPC models that express AR signaling components, including AR-V7. AR signaling is associated with pro-survival signaling. Both isolitsealiicolide C and isolinderanolide B reduced Bcl-2 protein expression. They also increased levels of cleaved PARP, a marker indicative of caspase-associated apoptotic cell death [57]. These findings demonstrate that the butanolides suppress AR/AR-V7 signaling and are associated with induction of apoptotic cell death [58,59]. These findings suggest that modulation of AR-associated pro-survival signaling may drive an apoptotic response to CRPC cells [60]. Additional studies are needed to define the downstream molecular events and determine whether the intrinsic mitochondrial apoptotic pathway is directly involved.

## 5. Limitation

A limitation of this study is that, through standardizing and validating a high-throughput, AR-V7-focused screening technology aimed toward the identification of potential agents for chemoprevention, comprehensive characterization of all active fractions and compounds is not achieved. As a result, only a subset of prioritized hits received detailed mechanistic and structural analyses, somewhat limiting the discovery of novel mechanisms and pharmacophores. Furthermore, the complete toxicity of the isolated butanolides toward normal prostate epithelial cells was not assessed due to the technology-development focus of this work and the likelihood that more potent molecules will be discovered through full-scale HTS. Future research will expand the platform to include a broader range of prostate cancer models, including AR-negative cell lines and a significantly larger number of pre-fractionated samples to increase the opportunity to discover potent, selective natural product modulators of this important target. It will also incorporate larger-scale isolation of prioritized lead compounds to enable more extensive pharmacological, toxicity, and translational characterization.

## Data Availability

The HRESIMS and NMR data for the natural products have been deposited in the Harvard Dataverse (dataverse.harvard.edu) at https://doi.org/10.7910/DVN/JNDOWK.

## Supplementary Materials

The following supporting information can be downloaded at https://www.mdpi.com/article/10.3390/pharmaceutics18091158/s1, Figure S1: Optimization of cell seeding density for resazurin-based viability assay in 384-wells platform; Figure S2: Optimization of Ideal Extract Concentrations to Evaluate Cytotoxicity on CRPC Cells.
