# Supplementary material for: Developing a Fluorescence-Based High-Throughput Screening Method for Natural Product Fractions to Identify Inhibitors of AR-V7 for Castration-Resistant Prostate Cancer
Source: Pharmaceutics. 2026 Sep 15;18(9):1158. doi: 10.3390/pharmaceutics18091158 (PMC13610743; doi:10.3390/pharmaceutics18091158)
Supplement: Supplementary file 1 [file pharmaceutics-18-01158-s001.zip › pharmaceutics-4495794-supplementary.pdf]

<u>Supplementary Figure S1 Optimization of cell seeding density for resazurin-based viability assay in 384-wells platform</u>

22Rv1 cell density (24h)

Cells/ml	500000	250000	125000	62500	31250	15625	7813	3906	1953	977	488	244	122	61	31	15	8	4	2	1	0	0	0	0
	1	2	3	4	5	6	7	8	9	10	11	12	13	14	15	16	17	18	19	20	21	22	23	24
A	59811	72000	74616	77542	78938	79361	68884	45950	38161	22031	16146	10697	10300	5670	5967	4990	4846	4725	5126	4767	4695	4639	4567	4117
B	65348	79847	79594	83072	81185	73538	59869	48133	31925	20782	12812	10595	7977	5645	6242	5360	5325	5214	5194	5182	5153	5089	5002	4572
C	67723	80432	84039	84917	82998	77051	68404	58581	43790	29478	19833	16632	10621	8317	6817	6137	6175	5428	5370	5256	5216	5234	5084	4661
D	67452	80586	83280	85334	84289	79612	70799	55874	40995	29619	26054	15401	10951	9181	7448	6417	5610	5379	5519	5429	5305	5274	5117	4769
E	69538	79705	80672	83424	81810	73476	56284	37775	26792	14880	13639	10892	7276	6360	5540	5325	5335	5359	5274	5242	5225	5209	5109	4708
F	68619	81773	84326	86021	84498	77781	69724	52505	38471	24509	16489	12845	10326	7209	5992	6687	5383	5480	5484	5326	5314	5252	5188	4769
G	70785	81386	83329	86243	83147	76542	63668	42972	31294	20480	16096	14159	9518	5763	5595	5533	5415	5382	5332	5334	5273	5250	5112	4787
H	70403	83049	85527	85447	83524	84178	70132	55083	39050	30067	19069	12871	10017	8007	7642	6341	5846	5647	5747	5642	5568	5522	5345	4978
I	60595	64812	68611	74422	79618	81474	72107	54677	39705	33332	21198	11970	9488	7345	6889	5243	5078	4971	4918	4915	4903	4828	4574	4225
J	59709	65659	70760	74793	80030	83077	80008	63739	50892	27162	19386	18547	10856	8038	6063	6183	5517	5102	5375	5129	5259	4819	4686	4235
K	61636	66840	69492	74406	78734	82817	81262	65835	55257	43634	27070	27397	15341	10454	7405	7103	6469	5550	5525	5388	5477	4740	4683	4275
L	60669	66561	71785	75834	80420	83127	80022	73892	52499	36283	29625	18596	13011	9475	6942	7335	5287	5720	5364	5042	4970	4850	4683	4253
M	61090	65492	69550	72567	79829	82323	78041	53954	39036	31331	16872	16876	8941	7504	7354	7408	5614	5654	4837	4980	4889	4724	4615	4265
N	61615	65821	71542	75419	81228	83147	75721	60620	48993	32867	22999	15086	10535	8634	6384	5860	5740	5567	5125	4993	4964	4833	4724	4242
O	58327	62545	67473	71924	76801	79016	75863	62207	48082	37886	24118	14839	16020	11465	7169	6003	5558	5578	4833	5046	4845	4614	4624	4110
P	51614	55666	60327	64327	69875	72167	71491	60488	43774	35588	28424	15539	12542	7496	9084	6208	5486	5368	4876	4672	4435	4315	4841	3655

22Rv1 cell density (48h)

	500000	250000	125000	62500	31250	15625	7813	3906	1953	977	488	244	122	61	31	15	8	4	2	1	0	0	0	0
	1	2	3	4	5	6	7	8	9	10	11	12	13	14	15	16	17	18	19	20	21	22	23	24
A	53215	66745	70106	73325	75283	78127	78262	71098	63557	42470	30913	19287	18873	8380	9257	6975	6790	6460	7463	6551	6414	6301	6094	5273
B	61198	77638	77755	81726	87059	88358	84228	76027	58404	40235	23540	19233	13621	8158	9802	7671	7554	7435	7261	7190	7151	7049	6879	6076
C	66011	78808	83132	85542	88374	89804	88923	85345	73340	56596	39721	33925	19932	14421	10968	9281	9782	7720	7603	7330	7332	7350	7037	6253
D	64945	78864	82027	86124	89481	90979	90074	83975	71143	56586	53355	30418	19768	16331	12542	10214	8206	7628	7852	7816	7469	7380	7127	6408
E	67086	78495	79505	86427	89413	90878	83816	67834	52316	29025	26841	20515	11724	9713	7992	7726	7567	7640	7431	7373	7402	7363	7119	6372
F	67070	80684	83766	87849	89375	91599	90617	81439	68561	48735	32962	24794	18777	11844	8818	10910	7641	7794	7915	7541	7510	7366	7219	6403
G	68487	80004	82453	86866	89934	91401	88509	73351	60002	42082	32856	28920	17964	8237	8060	7907	7761	7726	7527	7509	7502	7393	7177	6435
H	68480	82176	85803	88520	91888	96606	92666	83642	68947	58830	38467	24464	18266	13243	12710	9572	8686	8182	8137	7954	7881	7850	7553	6845
I	55532	60829	65339	72064	77816	82123	82033	80995	69956	61862	41428	23286	17310	12561	11002	7587	7423	7213	6985	7092	6964	6926	6369	5635
J	56512	62500	68622	73412	79912	83556	82642	85047	81082	52023	37849	37496	20562	14056	9435	9522	8237	7400	7985	7552	7631	6890	6642	5692
K	59099	63873	67153	73001	78126	83412	82074	84720	84377	75136	52600	53721	30923	20128	12889	11737	10609	8387	8389	8402	8335	6769	6678	5791
L	57192	63307	69636	74406	79477	83472	83247	85019	82799	66768	58478	37758	25122	17025	11774	12521	7876	8770	8112	7370	7134	6989	6605	5755
M	56455	62209	67237	70765	79547	82295	82628	82003	70096	59294	33403	34023	15820	13028	12346	13061	8617	8395	6873	7227	7051	6661	6486	5745
N	56723	62088	69002	73662	80538	82938	83847	84558	79929	60973	46276	31155	20072	15572	10182	9095	8523	8428	7512	7274	7031	6858	6570	5785
O	53403	58616	64528	69273	74434	77671	79225	80771	76493	66830	46321	28421	30883	22226	12099	9439	8641	8671	6906	7235	6938	6440	6435	5479
P	44845	49459	54730	58890	64088	66381	67928	68508	65803	58440	49390	28558	23153	12764	15724	9652	8228	7777	7084	6584	6066	5859	7114	4714

Supplementary Figure S1: *22RV1 cells were seeded across a gradient ranging from 500,000 to 0 cells/mL into the 384-well plate, incubated for 48 hours at 37°C with 5%* CO_2 *followed with DMSO treatment (1 μL/well) for 24 hours**(A)** and 48 hours**(B)**. Cell viability was assessed by adding a resazurin solution to each well, followed by incubation at 37°C for 16 hours. The fluorescence intensity was then measured at excitation/emission (Ex/Em) wavelengths of 560/590 nm. The change in color from green to red indicates a decrease in confluency; the optimal cell density is located at the transition zone. The top row (above the A1-24) indicates the number of cells/ml in the corresponding column.*

Supplementary Figure S2: Optimization of Ideal Extract Concentrations to Evaluate Cytotoxicity on CRPC Cells.

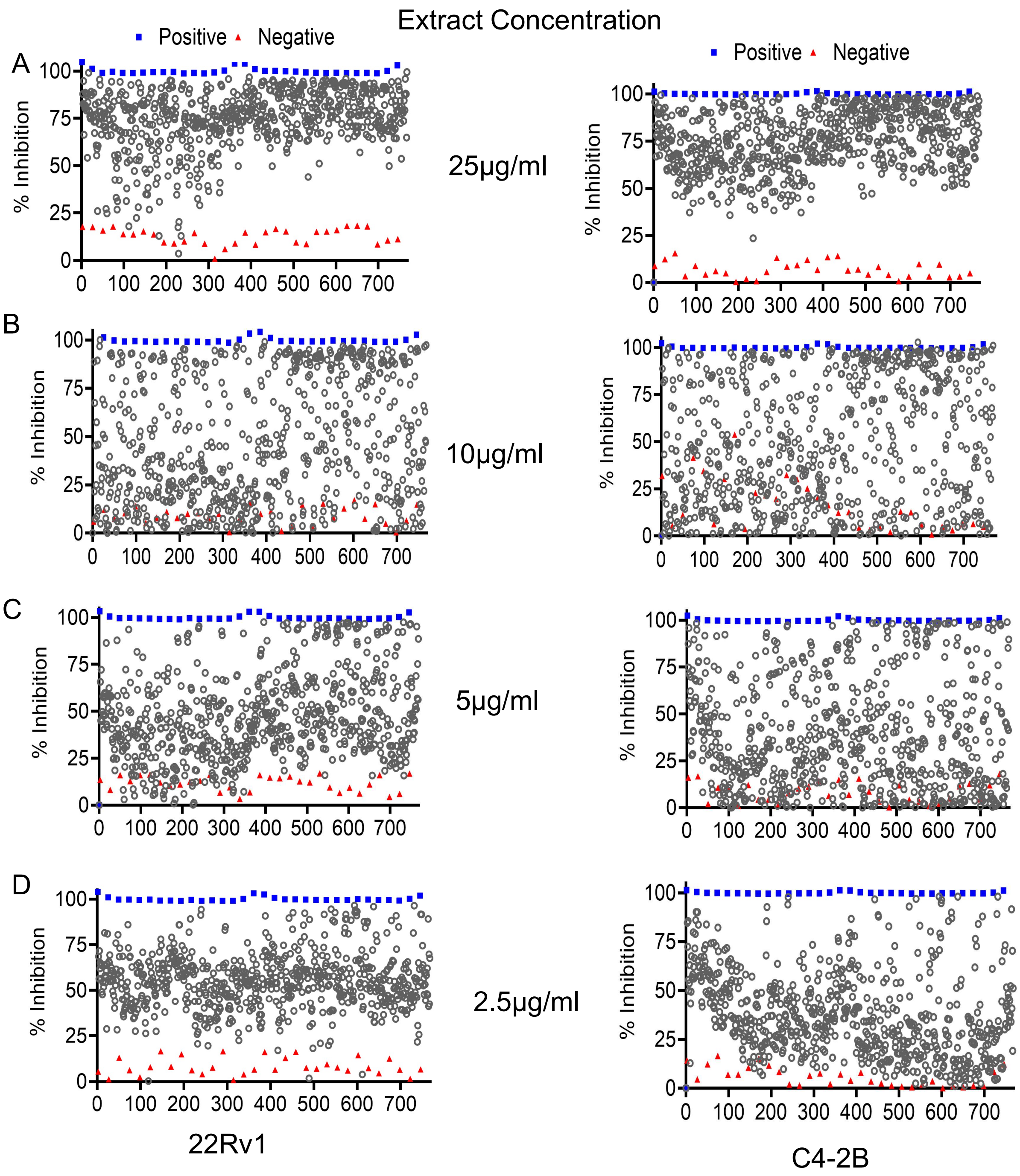

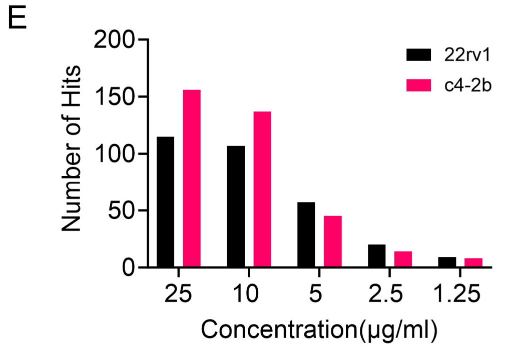

Suppl Figure S2: *(A–D) 22RV1 and C4-2B cells were treated with natural product extracts at specified concentrations (10 µg/mL, 5 µg/mL, 2.5 µg/mL, and 1.25 µg/mL), and cell viability was measured using an optimized resazurin protocol. The results were calculated and expressed as % Inhibition. In the analysis, blue indicates the positive control (WA at 15µM), while red represents the negative control (1% DMSO). Additionally, a graphical representation (I) illustrates the number of hits at different extract concentrations.*
